# The epidemiology of food allergy in primary care clinic: A cross-sectional study

**DOI:** 10.1097/MD.0000000000035641

**Published:** 2023-11-17

**Authors:** Dalal Al Hasan, Sara Al Hasan

**Affiliations:** a Emergency Training Center, Sharq, State of Kuwait; b Al-Adan Specialty Clinic, Ministry of Health, Shwaikh, State of Kuwait.

**Keywords:** allergen, angioedema, food allergy, hives, knowledge, Kuwait, primary care

## Abstract

Food allergy (FA) is an increasing global public health concern. Little is known about FA counsel in primary care clinics. The objective of this study is to describe the characteristics of FA in primary care clinics. It also aims to report the national primary care physicians’ current knowledge and practices. An electronic cross-sectional questionnaire was distributed to primary care physicians working at the Ministry of Health primary care clinics, across Kuwait’s’ 6 health districts, between May and June 2023. The questionnaire was made of 3 sections: participants’ demographic, FA counsel characteristics, participants’ knowledge and practices during FA counsel, and 37 variable tools. Eight-seven percent of primary care physicians counseled a patient with FA within the last 12 months. Most FA patients were children and infants. Approximately 2 out of 10 primary physicians counseled > 1 FA case/week. Prevalence of clinical presentation was: angioedema (23%), many skin hives (21%), few skin hives (19%), and mouth itch (9.4%). Prevalence of allergens was; peanuts (46%), shellfish (37%), eggs (36%), and tree nuts (36%), respectively. The mean of primary care physicians’ correct answers about FA was 58% and only 26% of primary care physicians acquired a sufficient amount of knowledge about FA, scoring above 67%. Their Knowledge scores about FA: clinical presentation 7 ± 1.6, diagnostic tests 2 ± 1, treatment 2.6 ± 1, and prevention 3 ± 1. In practice, correct treatment was offered by 30% of physicians, and 55% made the right referrals 86% are longing for training about FA. FA is a common counsel in primary care clinics. The most common FA presentation is a severe allergic reaction in the pediatric population. The current primary care physicians have insufficient knowledge about counseling FA and long for further training. Collectively, protocols and training for FA counseling should be launched in primary care.

## 1. Introduction

In allergy, a harmless substance is viewed by the immune system as an invader leading to an abnormal chain of reactions in the body. The substance can be an inhalant, chemical, sting, or even food.^[1–3]^ Food is a common allergen, and any type of food can cause an allergic reaction. Nine types of food cause 99% of food allergic reactions.^[4]^

Unfortunately, food allergy (FA) is an increasing global public health concern that affects children and adults.^[4,5]^ and Aside from food allergic reactions’ distressing signs and symptoms, rarely FA can lead to a life-threatening medical emergency called anaphylaxis (an-uh-fil-LAX-is). Anaphylaxis can progress in minutes, and it suddenly becomes hard for humans to breathe or pump blood. Every 3 minutes a patient with anaphylaxis arrives at the Emergency department (ED).^[4]^

Primary health care physicians are the first health care providers who assist patients with food allergic reactions. FA counsel represents 6% of their consultations.^[6]^ Yet, primary care physicians received little or no training concerning this disease area.^[7,8]^ Previous research about the characteristics of FA counsel in primary care is lacking. There is also evidence that primary care physicians have fair to poor knowledge about FA.^[9,10]^

Competent primary health care physician in FA counsel can reduce patient and family suffering and the burden of anaphylaxis on emergency department.^[11]^

The objective of this study is to describe the characteristics of FA in primary care clinics. It also aims to report the national primary care physician current knowledge and practices.

## 2. Method

### 2.1. Setting and design

Kuwait Ministry of Health (MOH) primary care clinics are distributed over 6 health districts: Al-Asimah (25), Hawali (16), Mubarak Al-Kabeer (16), Al-Ahmidi (23), Al-Farwanya (22), and Al-Jahra (14).

There is no specific protocol for FA management in these primary care clinics, however the clinics have anaphylaxis management and allergy referrals protocols (Appendix 1, Supplemental Digital Content, http://links.lww.com/MD/K772 and 2, Supplemental Digital Content, http://links.lww.com/MD/K773).

Across sectional electronic questionnaire was distributed over primary care physicians in the 6 health districts via emails between May 6th and June 6th 2023. Emails were retrieved from primary care clinics directors or their representatives. The email recipients were invited to voluntarily complete the anonymous survey. Completion of the questionnaire implied consent to participate. It took about 10 minutes to complete the questionnaire and no compensation was given to questionnaire respondents for completing it.

### 2.2. Instrument development

Although primary care clinics do not have a specific protocol for managing food allergies, they do have protocols for managing anaphylaxis and referring patients to an allergist, which can be found in Appendix 1, Supplemental Digital Content, http://links.lww.com/MD/K772 and 2, Supplemental Digital Content, http://links.lww.com/MD/K773. To gather information, a cross-sectional electronic questionnaire was distributed via email to primary care physicians in all 6 health districts between May 6th and June 6th, 2023. The email addresses were obtained from primary care clinic directors or their representatives. Participation in the survey was voluntary and anonymous, and completion of the questionnaire indicated consent to participate. The survey took approximately 10 minutes to complete, and no compensation was offered to respondents.

The questionnaire was divided in to 3 sections: participant’s demographics, FA counsel characteristics and participant’s current practices and knowledge. The final questionnaire included 37 variables.

Gender, age, health district, and years of experience were the 4 demographic variables were. Participants were then asked if they counseled any patients with FA in the last 12 months.

Participants who responded with a “Yes” were prompted to provide details about the FA council’s attributes, such as the types of food, clinical manifestations, diagnostic measures, treatment options, and referrals. Alternatively, those who replied with a “No” were asked to complete a true/false section to evaluate their comprehension of food allergies. This section consisted of questions regarding clinical presentation, diagnosis, treatment, and prevention, with 10, 5, 5, and 6 questions, respectively. All queries in the knowledge segment were based on the most up-to-date recommendations from Food Allergy Research and Education and the American Academy of Allergy, Asthma, and Immunology with regard to FA diagnosis, treatment, and prevention.^[12–18]^

As part of the study, primary care physicians were awarded points for each correct answer in 4 subsections: clinical presentation, diagnosis, treatment, and prevention. Wrong answers received zero points. To determine a physician’s overall knowledge score regarding FA, points were totaled across all subsections. Participants who scored at least 67% (answering two-thirds of the questionnaire questions correctly) were considered knowledgeable. For a detailed description of the study questionnaire and its variables, please refer to Material 1, Supplemental Digital Content, http://links.lww.com/MD/K774.

To optimize user-friendliness and thus response rate the questionnaire was available in English. To avoid compromised validity and content clearance the author structured the questionnaire and a primary care physician reviewed it. The researcher also evaluated the questionnaire reliability using the initial 10 responses and Cronbach alpha α was kept equal to 0.84, this ensured the internal consistency of the questionnaire.

### 2.3. Participants

Only primary care physicians working in MOH primary care clinics were included in the analysis.

### 2.4. Sample size

Convenient sampling was used in this study. All practicing primary care physicians across the 6 health districts who agreed to answer the questionnaire were included in the analysis.

The primary outcome is primary care physician knowledge about FA. In which. Primary care physicians are categorized as knowledgeable if their total score was at least 67% and not knowledgeable if the they score less than 67%.

### 2.5. Ethical approval

The study received IRB approval from the Kuwait MOH independent ethics committee on 5 June 2023 (No. 1951). No administrative permission were need to acquire the collected data. All methods were performed in accordance with the relevant guidelines and regulations or declaration of *Helsinki*.

### 2.6. Statistical method

Statistical analysis will be performed using Excel (version 23 for Windows) and Statistical Package for Social sciences (SPSS Version 23; IBM, NY) descriptive analysis was performed to determine frequencies, percentages and means of primary care physicians demographics, FA counsels characteristics and primary care physicians level of knowledge. The results will be expressed in mean ± standard variation. The value of *P* < .05 will be considered statically significant. Linear regression will be used to associate factor with primary care physicians’ knowledge. This is to identify which factor primary care physicians’ knowledge. all data will be handled in a secure password-locked computer. Data will be monitored on an ongoing basis for completeness and precision by the data manager.

## 3. Results

Ninety-five practicing primary care physicians were included in the analysis from MOH primary care clinics, across Kuwait’s 6 health districts. Eighty-seven percent of the primary care physicians reported counseling a FA patient in their clinics during the last 12 months.

Participating primary care physicians’ demographics is illustrated in Table 1.

**Table 1:** Primary care physician demographics in Ministry of Health primary care clinics, 2023.

Variable	N = 95 (%)
Gender	
Male	30 (32)
Female	65 (68)
Age (median ± SD)	40 ± 35
Years of experience (mean ± SD)	11 ± 8
Health care district	
Al-Asemah	33 (35)
Hawali	3 (3)
Mubarak Alkabeer	32 (34)
Al-Ahmidi	14 (15)
AL-Farwanya	11 (12)
Al-jahra	2 (2)

In terms of the FA counsel characteristics, approximately 2 out of 10 primary care physicians counsel > 1 patient per week in their clinics for FA.

The majority of FA patients were from the pediatric population (61%) (Table 2).

**Table 2:** Food allergy counsel characteristics in Ministry of Health primary care clinics, 2023.

Variable	N = 83 (%)
Age group of food allergy patients	
Adult	25 (26)
Pediatric	58 (61)
Frequency of food allergy counsel in primary care clinic	
>1 a week	17 (18)
1 every week	3 (3)
1 every month	15 (16)
1 every 3–6 mo	35 (36)
1 every 6–12 mo	21 (22)
Type of food as trigger of the allergic reaction	
Eggs	34 (36)
Peanuts	44 (46)
Tree nuts	34 (36)
Milk	11 (11)
Fish	28 (30)
Shellfish	35 (37)
Soy	3 (3)
Sesame	12 (13)
Fruits	24 (25)
Wheat	0 (0)
Type of allergic reaction	
Mild allergic reaction	28 (29)
Severe allergic reaction	26 (27)
Both mild and severe allergic reactions	29 (30)

**Figure 1.**
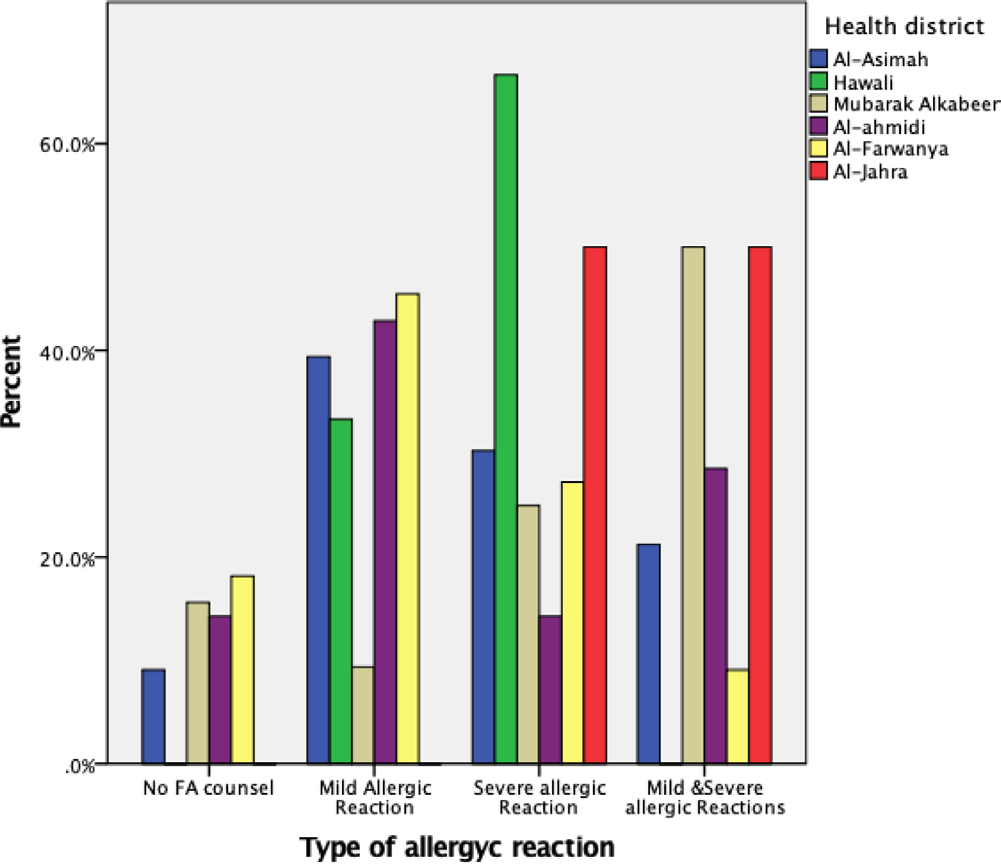
Type of allergic reaction per Kuwait Health District, 2023.

In relation to severity, both mild and severe food allergic reactions were counseled in clinics over the last year. However, Hawali health district had high rates of severe food allergic reactions (Fig. 1).

**Figure 2.**
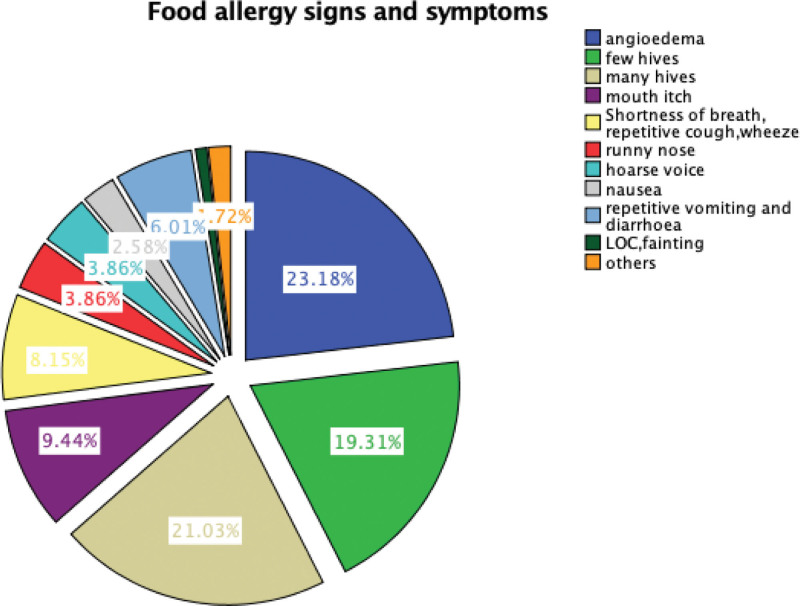
FA clinical presentation in Ministry of Health primary care clinics, 2023. FA = food allergy.

Most common clinical presentations of FA were angioedema (23%), many skin hives (21%), and few skin hives (19%), respectively (Fig. 2). Prevalence of allergens was; peanuts (46%), shellfish (37%), eggs (36%), and tree nuts(36%) respectively.

In terms of primary physicians’ knowledge and practices during FA counsels. Only 26% of primary care physicians acquire sufficient amount of knowledge about FA. Knowledge strength was recorded in FA clinical presentation scores 7 ± 1.6. While weaknesses were documented in diagnostic tests, treatment, and prevention scores in the knowledge-based questions (Table 3).

**Table 3:** Primary care level of knowledge about food allergy in Ministry of Health primary care clinics, 2023.

Variable	N = 95 (%)
Total clinical presentation score (out of 10 points), (mean ± SD)	7 ± 1.6
Total diagnostic test score (out of 5 points), (mean ± SD)	2 ± 1
Total treatment score (out of 5 points), (mean ± SD)	2.6 ± 1
Total prevention score (out of 6 points), (mean ± SD)	3 ± 1
Total level of knowledge (out of 26 points), (mean ± SD)	15 ± 3
Level of knowledge (%)	58
Interest in FA training	82 (86)

FA = food allergy.

One factor was associated with primary physicians level of knowledge, patients’ age group (*P* = .017, 0.15 [95% CI: −2, −0.048]) (Table 4).

**Table 4:** Influence of food allergy counsel characteristics and primary care physician demographics on primary care physician knowledge about food allergy, using logistic regression.

Variable	*P* value	OR (95% CI)
Age	.23	0.13 (−0.27, 0.04)
Years of experience	.628	0.02 (−0.05, 0.27)
FA patients age group (pediatric)	.017	0.15 (−2, −0.048)
Type of allergic reaction severe	.047	1.5 (0.071, 4.4)

CI = confidence interval, FA = food allergy, OR = odds ratio.

Having said that in practice, 30% of primary care physicians have appropriately treated FA patient and 55% referred them to the appropriate health care (Table 5).

**Table 5:** Primary care physicians’ practices during food allergy counsel in Ministry of Health primary care clinics, 2023.

Variable	N = 83 (%)
Treatment within guideline recommendation	30 (31)
Diagnostic tests within guideline recommendations	7 (7)
Referral within guideline recommendations	52 (55)

## 4. Discussion

This study provides new insights into the characteristics of FA counsel in primary care clinics in the Arabian Gulf region. The research evaluates the knowledge and practices of primary care physicians during FA counsel. Similar to existing literature, the rates of FA counsel in Kuwait’s primary care clinics are high. Gupta et al^[9]^ reported that 99% of family medicine in the U.S, counsel FA patient in their clinics. Moreover, the pediatric population counseled primary care clinics for FA more than adults. These findings are again consistent with the current literature, according to U.S. Center for Diseases Control & Prevention, FA is more prevalent in children than adults.^[4,19]^ And although the overall food types that trigger allergic reaction in Kuwait are similar to those in the US, the prevalence array is different.^[20]^ Peanuts, shellfish, eggs and tree nuts are the most common food allergen respectively. Whereas in the U.S. milk, eggs followed by peanut are the most common food allergens respectively.^[20]^

To the authors knowledge this is the first study to report clinical presentation, type of allergic reaction and frequency of FA counsel in primary care clinics. Identifying locations of primary care clinics with frequent and or severe allergic reactions can help policy makers in preparing clinics to manage patients with FA. This can reduce the suffering of patients and the burden of acute anaphylaxis on hospital ED.

Very common clinical presentations of FA were angioedema, many hives, few hives and respiratory symptoms respectively. These findings are addendum to literature.

One ED retrospective study reported different clinical presentations. In order; hives, anaphylaxis, respiratory distress were the most frequent FA presentations in ED.^[21]^

In respect to participants demographics, our primary care physicians’ demographics analogous those in literature.^[10]^ Additionally, their level of knowledge about FA counsel, 58% is comparable to those reported in current research, 47% to 61%.^[9,10]^

Interestingly weaknesses in knowledge about diagnostic tests seems like a common problem between primary care physicians, as similar findings were reported by both studies in the US and Turkey.^[9,10]^

Weaknesses in treatment and prevention is partially in line with present research, Al-Herz et al^[22^ national study on pediatricians’ awareness about FA recorded fair pediatricians’ knowledge about FA treatment, 77% but low knowledge levels in prevention, 42%.

At last few factors influenced primary physician level of knowledge with the most prominent, patients’ age group.

Physicians in Kuwait just like those in Turkey longing for training about FA. The level of interest in receiving in training in Kuwait is 86%, whereas in Turkey, 98.2%.^[10]^

To this end, our physicians’ practice in primary care clinic resembles others in referral rates.^[9]^

Number of limitations should be acknowledged. Voluntary participation and self-reporting were potential sources of bias. Bias can be eliminated with random participation. Furthermore, the FA counsel characteristics was based on primary physicians’ recall. This again made the study open to reporting bias. The current research maximized the optimum recall period by asking participants to record events within the last 12 months. the results of this study can be generalized over the Arabian gulf region. Other countries have different health care system and population demography in which replication of this study in another setting can produce different results.

## 5. Conclusion

FA is a common counsel in primary care clinics. The most common FA presentation is severe allergic reaction in the pediatric population. The current primary care physicians have insufficient knowledge about counseling FA and longing for further training. Collectively, protocols and training for FA counsels should be launched in primary care.

## Acknowledgments

The authors would like to acknowledge and thank Emergency Training Center for the enormous help and support rendered in the course of designing this study.

## Author contributions

**Conceptualization:** Dalal Al Hasan.

**Data curation:** Dalal Al Hasan, Sara Al Hasan.

**Formal analysis:** Dalal Al Hasan.

**Investigation:** Dalal Al Hasan.

**Methodology:** Dalal Al Hasan.

**Project administration:** Dalal Al Hasan.

**Resources:** Dalal Al Hasan.

**Software:** Dalal Al Hasan.

**Supervision:** Dalal Al Hasan.

**Validation:** Dalal Al Hasan.

**Visualization:** Dalal Al Hasan.

**Writing – original draft:** Dalal Al Hasan.

**Writing – review & editing:** Dalal Al Hasan.

## Supplementary Material






