# Supplementary figures and images for: The epidemiology of food allergy in primary care clinic: A cross-sectional study
Source: Medicine (Baltimore). 2023 Nov 17;102(46):e35641. doi: 10.1097/MD.0000000000035641 (PMC10659696; doi:10.1097/MD.0000000000035641)

**
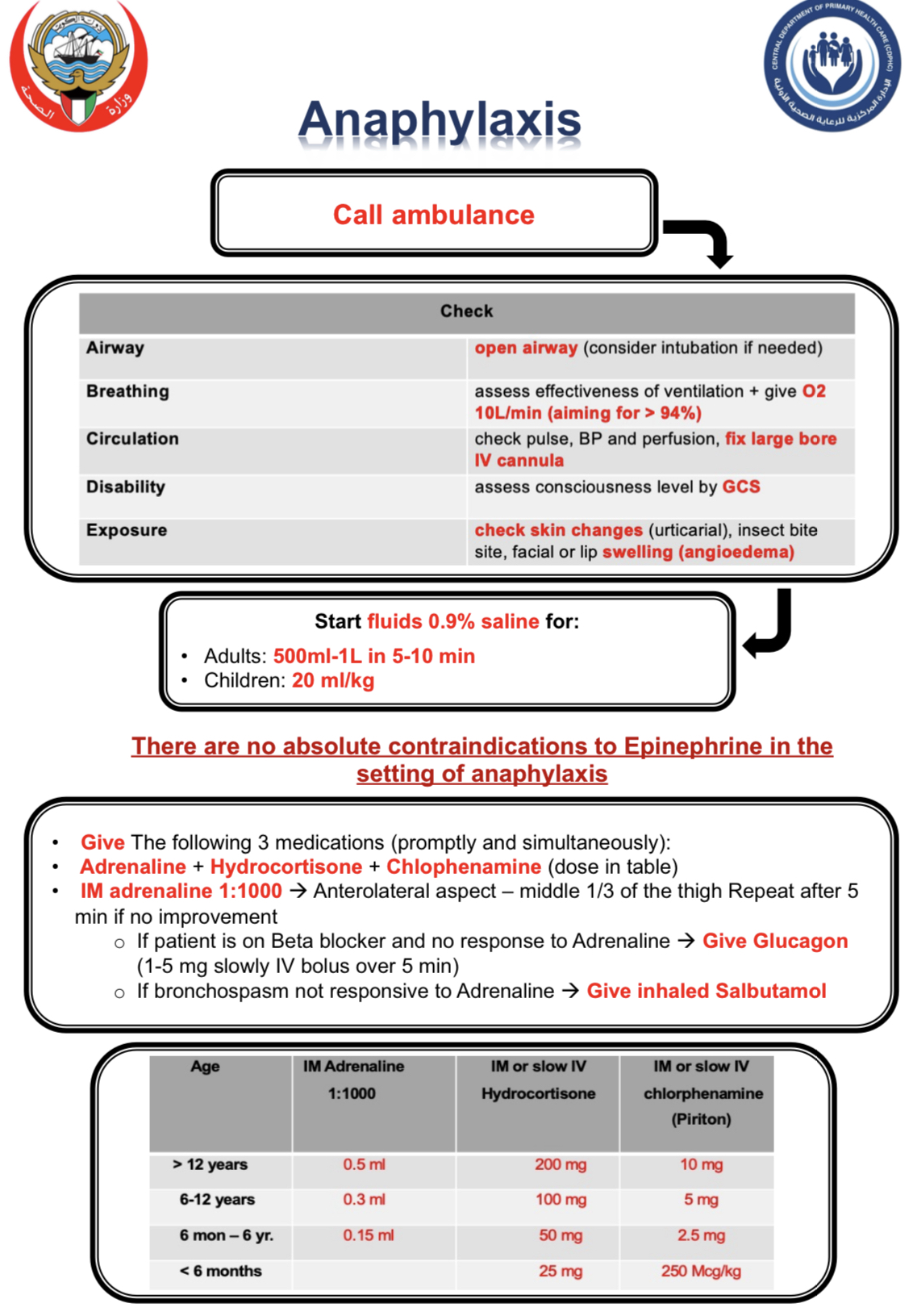
**

Supplement: Supplementary file 1 [file medi-102-e35641-s001.docx]

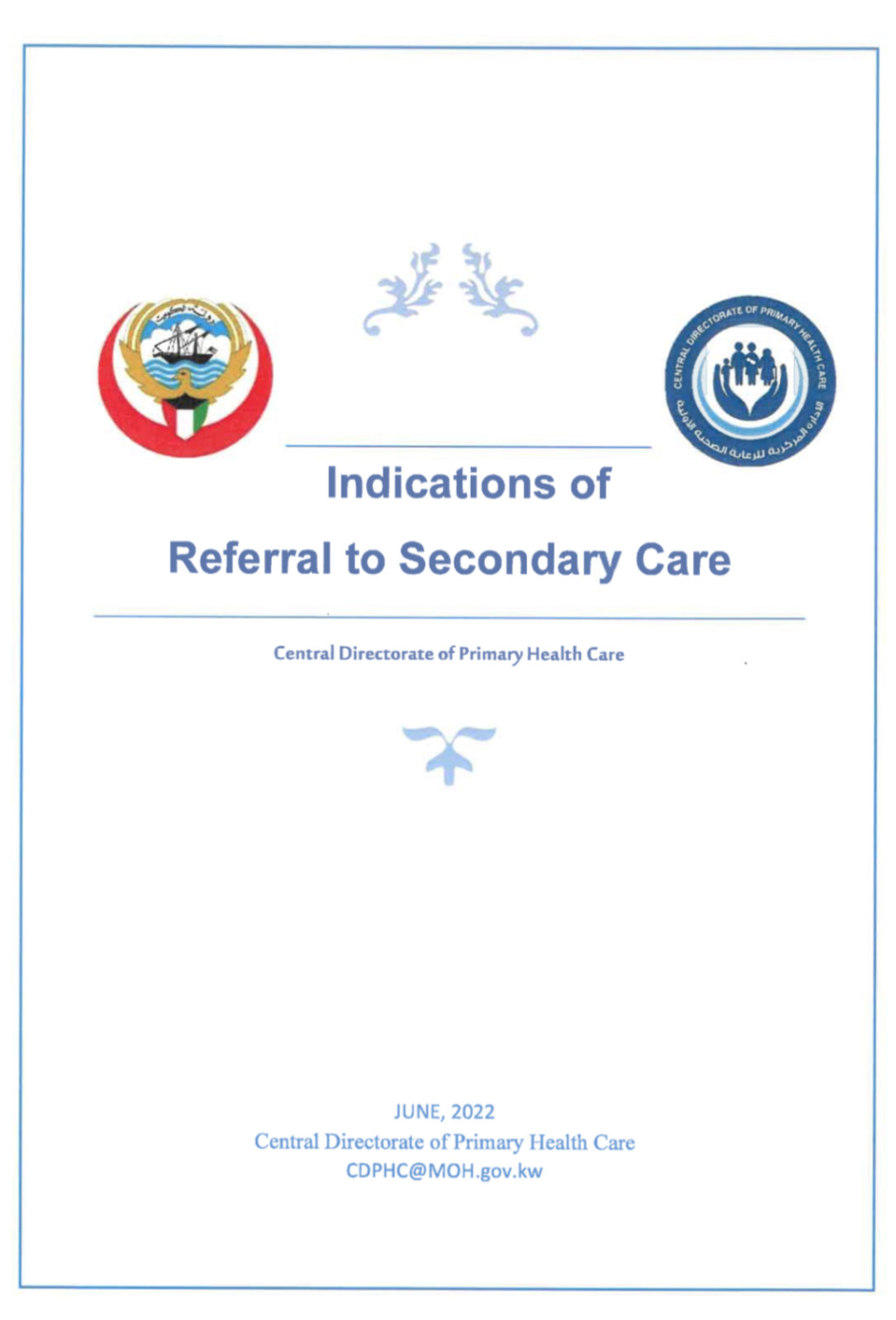

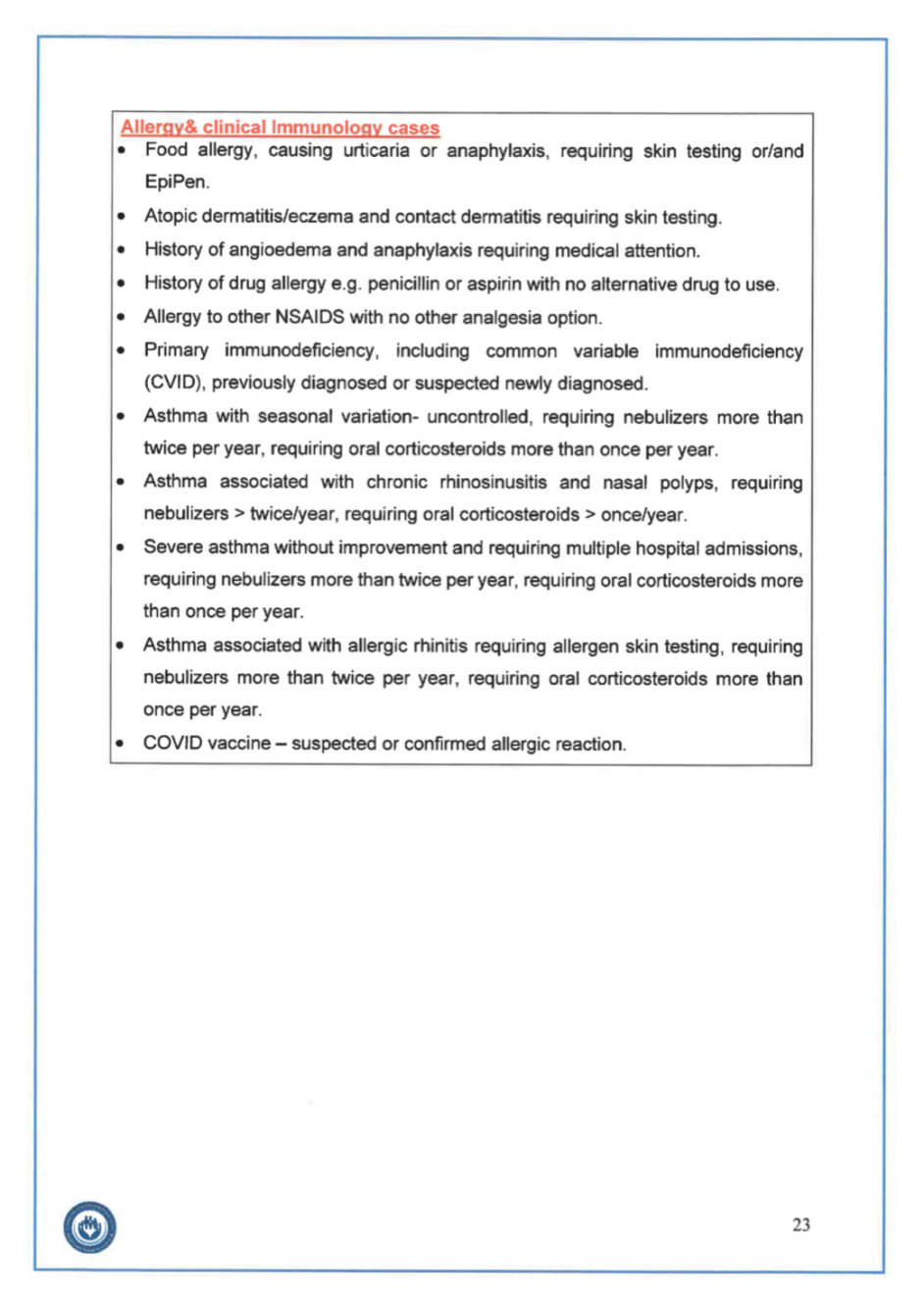

Supplement: Supplementary file 2 [file medi-102-e35641-s002.docx]
