# Supplementary material for: The epidemiology of food allergy in primary care clinic: A cross-sectional study
Source: Medicine (Baltimore). 2023 Nov 17;102(46):e35641. doi: 10.1097/MD.0000000000035641 (PMC10659696; doi:10.1097/MD.0000000000035641)
Supplement: Supplementary file 3 [file medi-102-e35641-s003.docx]

**Supplemental appendix 1. Detailed description of the study questionnaire.**

**TITLE: FOOD ALLERGIES IN PRIMARY CARE QUESTIONNAIRE**

**Section 1 : Participants demographics**

*1. Age* [FREE TXT]

*2. Gender* [MULTIPLE CHOICE QUESTION]

a. Male

b.Female

*3. Years of working in primary care clinics (years of experience)* [free text]

*4. Primary care center health district:* [MULTIPLE CHOICE QUESTION]

a. Al-Asimah

b. Hawali

c. Mubarak Al-Kabeer

d. Al-Ahmidi

e. Al-Farwanya

f. Al-Jahra

*5. Have you seen a patient with food allergy in your clinic with in the last 12 months?* [MULTIPLE CHOICE QUESTION]

a. Yes [directed to section 2]

b. No [directed to section 3]

**Section two: FA counsel characteristics**

*Q1. What was/were the age group of the FA patients?* [MULTIPLE CHOICE QUESTION]

a. Adults

b. pediatirc patients: Children (1-8 years) & infant (1 month- 1 year)

c. Adults and pediatric patients/

*Q2. How frequently do you see FA cases in your clinic have you seen in the last 12 months?* [MULTIPLE CHOICE QUESTION]

a. > one FA case every week

b. One FA case every week

c. One FA case every month

d. One FA case every 3-6months

e. One FA case every 6-12 months

*Q3. What was the trigger of most FA cases? Choose more than 1 if needed* [MULTIPLE CHOICE QUESTION]

a.Wheat

b.Eggs

c. Peanuts

d. Tree nuts

e. Milk

f. Fish

g. Shellfish (shrimp,crabs etc)

h. Soy

i. Sesames

j. Fruits

k. Vegetables

Q4.*What was/were the clinical signs and symptoms you observed on these FA patients during your practice? Choose more than 1 if needed* [MULTIPLE CHOICE QUESTION] *

a. Runny nose

b. Mouth itch

c. Nausea

d. Few skin hives

e. Many skin hives

f. Swelling of lips or eyelids.

g. Hoarse voice

h. Shortness of breath or wheeze or repetitive cough

i. Fainting, loss of conscious ness

j. Repetitive diarrhoea or vomiting

k. Other

*Note: *The selection of these signs and symptoms were based on FARE* ***mild/severe allergic reaction*** *criteria. Primary physicians who only selected one or all of the following: few skin hives, mouth itch, nausea, runny nose were highlighted as* ***mild allergic reaction****. Primary physicians who only selected one or all of the following: many skin hives, swelling (angioedema), repetitive vomiting or diarrhoea, respiratory symptoms: shortness of breath, repetitive cough or wheeze, hoarse voice, fainting, loss of consciousness were highlighted as* ***severe allergic reaction. If the*** *Primary physicians selected both symptoms of mild and severe criteria the allergic reaction type was categorised as seen both mild and severe allergic reaction.*

Reference:

1.Food Allergy Research And Education. FARE Your Guide To Food Allergy; Newly Diagnosed With Food Allergy? We Can Help. Food Allergy Research And Education. United States.2020.[Online] Available from URL:< https://www.foodallergy.org/living-food-allergies/information-you/newly-diagnosed > [Last accessed on March 30 2023]

*Q5. How did you treat the FA case? Choose more than 1 if needed* [MULTIPLE CHOICE QUESTION] *

a.Antihistamine

b.Antihistamine and steroids

c.Antihistamine and epinephrine

d.Support ABC, epinephrine.

e.Nothing only referred the patient to ED

**Note: option a,c,d were categorised within the guideline recommendations. Option b &e were categorised* ***not with*** *in the guideline recommendations.*

*Reference:*

*1. American Academy of Asthma Allergy & Immunology. Insurance Coverage for H1-Antihistamines: Implications for Quality Healthcare and Public Safety. American Academy of Asthma Allergy & Immunology. United states.2002 [Online] Available from URL:<* [*https://www.aaaai.org/Aaaai/media/Media-Library-PDFs/Allergist%20Resources/Statements%20and%20Practice%20Parameters/Insurance-coverage-for-H1-antihistamines-2002.pdf*](https://www.aaaai.org/Aaaai/media/Media-Library-PDFs/Allergist%20Resources/Statements%20and%20Practice%20Parameters/Insurance-coverage-for-H1-antihistamines-2002.pdf)*> [Last accessed on May 23 2023]*

*2.Food Allergy Research And Education. Steroid role. Food Allergy Research And Education. United States. 2023. [Online] Available from* [*URL:<https://www.foodallergy.org/resources/recognizing-and-treating-reaction-symptoms#:~:text=Steroids%20may%20be%20given%2C%20typically,severe%20reaction%20from%20coming%20bac*](URL:%3chttps://www.foodallergy.org/resources/recognizing-and-treating-reaction-symptoms#:~:text=Steroids%20may%20be%20given%2C%20typically,severe%20reaction%20from%20coming%20bac)*> [Last accessed on June 3 2023]*

*Q6. Did you do any laboratory test? needed* [MULTIPLE CHOICE QUESTION] *

a.Yes. CBC, U&E.

b.Yes. CBC,U&E, Serum IgE

c.Yes. CBC,U&E, Serum IgE,IgG.

d.No. I did not do any laboratory tests.

**Note: option b was categorised within the guideline recommendations. All other Options were categorised* ***not with*** *in the guideline recommendations.*

*Reference:* Food allergy research and education. Food allergy diagnosis and testing. Food allergy research and education. United States. 2023. [Online] Available from URL:< <https://www.foodallergy.org/research-innovation/accelerating-innovation/food-allergy-diagnosis-and-testing>> [Last accessed on May 23 2023]

Q7*.Did you refer the FA patient?*

a. Yes. Paediatrician

b. Yes. Dermatologist

c. Yes. Alrashed Asthma and Allergy Center

d. Yes.Emergency department

e. No I didn’t refer him.

**Note: option a,c,d was categorised within the guideline recommendations. All other Options were categorised* ***not with*** *in the guideline recommendations. Based on MOH referral protocols appendix A&B.*

**Section Three: Primary care physician knowledge about FA (26 points) [true/false] each question 1 point.**

Subsection one: Clinical presentation (10 points)

1.*Cow’s milk protein allergy is always accompanied by bloody stools** [FALSE]

**Reference: American Academy of Asthma Allergy & Immunology. Food Protein-Induced Enterocolitis Syndrome (FPIES). American Academy of Asthma Allergy & Immunology. United states.2023 [Online] Available from* [*URL:<*](URL:%3c) [*https://acaai.org/allergies/allergic-conditions/food/food-protein-induced-enterocolitis-syndrome-fpies/*](https://acaai.org/allergies/allergic-conditions/food/food-protein-induced-enterocolitis-syndrome-fpies/)*> [Last accessed on June 3 2023]*

2. *Food allergic reaction always involves the skin*. [FALSE]**

*3.Mild allergic reaction is when there is one of the following symptoms, few hives, runny nose, mouth itchiness, nausea.* [True]**

*4.Mild food allergic reactions are self-limiting and do not need laboratory investigation or follow up. [FALSE]***

*5.Mild food allergic reaction is good sign, it means that this patient wont have severe food allergic reactions in the future to this trigger.* [FALSE]**

*6.Anaphylaxis should be only diagnosed if there is cardiac or respiratory symptoms.* [FALSE]**

***Reference: Food allergy research and education. Recognizing and Treating Reaction Symptoms. Food allergy research and education. United States. 2023. [Online] Available from URL:<* [*https://www.foodallergy.org/resources/recognizing-and-treating-reaction-symptoms*](https://www.foodallergy.org/resources/recognizing-and-treating-reaction-symptoms) *> [Last accessed on May 23 2023]*

7.*The first food allergic reaction can be followed by re-occurance hours after the allergic reaction.* [TURE]*

# *Reference: Food allergy research and education.* Reactions Are More Likely When Symptoms of Initial Reaction Are Severe. *Food allergy research and education. United States. 2023. [Online] Available from URL<* *https://www.foodallergy.org/fare-blog/biphasic-reactions-are-more-likely-when-symptoms-initial-reaction-are-severe > [Last accessed on May 23 2023]*

8*. If the patient is allergic to one type of tree nuts, then he is allregic to all types of nuts.* [FALSE]*

**Reference: Food allergy research and education* Reactions Tree nut allergy. *Food allergy research and education. United States. 2023. [Online] Available from URL<* https://www.foodallergy.org/living-food-allergies/food-allergy-essentials/common-allergens/tree-nut*> [Last accessed on May 23 2023]*

9. *There is no link between having hay fever and being allergic to orange.* [FALSE]*

**Reference: American Academy of Asthma Allergy & Immunology. Oral allergy Syndrome. American Academy of Asthma Allergy & Immunology. United states.2023 [Online] Available from URL:< https://www.aaaai.org/tools-for-the-public/conditions-library/allergies/oral-allergy-syndrome-(oas) > [Last accessed on June 3 2023]*

10. *Allergic reaction is dose dependent, the more the patient consume from the trigger the worse the reaction will be.* [FALSE]*

*Reference: *Food allergy research and education*. Food allergy myths and misconceptions. *Food allergy research and education. United States. 2023. [Online] Available from URL<* https://www.foodallergy.org/resources/food-allergy-myths-and-misconceptions*> [Last accessed on May 23 2023]*

**Subsection two: Diagnostic tests (5 points)**

1. *Skin prick testing is highly sensitive but only moderately specific.* [FALSE]*

*2.Food allergic serum IgE antibodies test is highly sensitive and specific.* [FALSE]*

*3.Food allergic Serum IgE is not useful for children with severe atopic dermatitis.* [FALSE]*

*4.Food-specific immunoglobulin G (IgG) and IgG4 tests are additional useful tests for allergies.* [FALSE]*

*5.Eosinophilia is an important/common finding in patients with a food allergy.* [FALSE]*

**Reference: Food allergy research and education. Food allergy diagnosis and testing. Food allergy research and education. United States. 2023. [Online] Available from URL:<* [*https://www.foodallergy.org/research-innovation/accelerating-innovation/food-allergy-diagnosis-and-testing*](https://www.foodallergy.org/research-innovation/accelerating-innovation/food-allergy-diagnosis-and-testing)*> [Last accessed on May 23 2023]*

**Subsection Three: Treatment (5 points)**

1.*The prophylactic use of an anti-histamine prevents anaphylaxis in food allergic patients.* [FALSE]*

*2.Cow’s milk allergic patients should be given goat’s or sheep’s milk to avoid nutritional deficiency. [*FALSE]*

*3.If antihistamine fails to control the mild allergic reaction, Steroids is the second line treatment.* [FALSE]*

*4.Antihistamine is the mainstay treatment for mild allergic reaction and should be given for 3 days only.* [FALSE]*

*5. For infants <6 months the mainstay treatment for severe food allergic reaction is supportive care as epinephrine cannot be given.* [FALSE]*

*6. If one type of antihistamine fails to treat the mild food allergic reaction, another type should be tried.* [True]*

References:

1.*American Academy of Asthma Allergy & Immunology. Insurance Coverage for H1-Antihistamines: Implications for Quality Healthcare and Public Safety. American Academy of Asthma Allergy & Immunology. United states.2002 [Online] Available from URL:<* [*https://www.aaaai.org/Aaaai/media/Media-Library-PDFs/Allergist%20Resources/Statements%20and%20Practice%20Parameters/Insurance-coverage-for-H1-antihistamines-2002.pdf*](https://www.aaaai.org/Aaaai/media/Media-Library-PDFs/Allergist%20Resources/Statements%20and%20Practice%20Parameters/Insurance-coverage-for-H1-antihistamines-2002.pdf)*> [Last accessed on May 23 2023]*

2. *Reference: Food allergy research and education. Milk allergy. United states.2023 [Online] Available from URL:< https://www.foodallergy.org/living-food-allergies/food-allergy-essentials/common-allergens/milk> [Last accessed on May 23 2023]*

*3.Shaker M, Wallace D, Golden D, et al. Anaphylaxis—a 2020 practice parameter update, systematic review, and Grading of Recommendations, Assessment, Development and Evaluation (GRADE) analysis. Journal of allergy & clinical immunology.2020; 145(4): 1082-1122.*

*4. Food Allergy Research And Education. Steroid role. Food Allergy Research And Education. United States. 2023. [Online] Available from* [*URL:<https://www.foodallergy.org/resources/recognizing-and-treating-reaction-symptoms#:~:text=Steroids%20may%20be%20given%2C%20typically,severe%20reaction%20from%20coming%20bac*](URL:%3chttps://www.foodallergy.org/resources/recognizing-and-treating-reaction-symptoms#:~:text=Steroids%20may%20be%20given%2C%20typically,severe%20reaction%20from%20coming%20bac)*> [Last accessed on June 3 2023]*

**Subsection four: Prevention (6 points)**

1.*If the patient is allergic to fruit or vegetable, he/she will be allergic to it in all its’ cooking forms; raw or cooked.* [FALSE]*

**Reference: American Academy of Asthma Allergy & Immunology. Oral allergy Syndrome. American Academy of Asthma Allergy & Immunology. United states.2023 [Online] Available from URL:< https://www.aaaai.org/tools-for-the-public/conditions-library/allergies/oral-allergy-syndrome-(oas) > [Last accessed on June 3 2023]*

2. *Allergic reaction to food is stimulated by eating the allergen only.* [FALSE]*

**Reference: Food allergy research and education. tips for keeping a safe home. United states.2002 [Online] Available from URL:< https://www.foodallergy.org/resources/tips-keeping-safe-home> [Last accessed on May 23 2023]*

3.*The use of hypoallergic milk reduce the risk of developing food allergy*. [FALSE]*

* Reference: *American Academy of Asthma* Allergy & Immunology. Prevention of Allergies and Asthma in Children. American Academy of Asthma Allergy & Immunology. United states.2023 [Online] Available from URL https://www.aaaai.org/tools-for-the-public/conditions-library/allergies/prevention-of-allergies-and-asthma-in-children > [Last accessed *on June 3 2023]*

4.*The use of soy milk is encouraged for infants with cow milk protein allergy.* [FALSE]*

** Reference: Food allergy research and education. Milk allergy. United states.2023 [Online] Available from URL:< https://www.foodallergy.org/living-food-allergies/food-allergy-essentials/common-allergens/milk> [Last accessed on May 23 2023]*

5. *Eggs should be fed after 12 months of age to reduce the risk of developing allergy to eggs.* [FALSE]*

**Reference: Food allergy research and education. Early introduction for other top allergens. United states.2023 [Online] Available from URL;< https://www.foodallergy.org/resources/early-introduction-other-top-allergens > [Last accessed on May 23 2023]*

6. *Breast fed babies should never drink cow milk before 6 months and that should reduce risk of cow milk allergy.* [FASLE]*

*Reference: *American Academy of Asthma Allergy & Immunology.* **Early introduction of infant formula to prevent cow’s milk allergy**. *American Academy of Asthma Allergy & Immunology. United states.2023 [Online] Available from URL<* *https://www.aaaai.org/Tools-for-the-Public/Latest-Research-Summaries/The-Journal-of-Allergy-and-Clinical-Immunology/2020/formula> [Last accessed on June 3 2023]*

**Training interest: [MULTIPLE CHOICE QUESTION]**

*1.I am interested in receiving up to date information or training regarding food allergy counselling.*

a. Yes

b. No
